# Efficacy of acupuncture treatment for diarrhea-predominant irritable bowel syndrome with comorbid anxiety and depression: A meta-analysis and systematic review

**DOI:** 10.1097/MD.0000000000040207

**Published:** 2024-11-15

**Authors:** Zhen Wang, Yi Hou, Hongwei Sun, Zhengwen Wang, Haiyan Zhang

**Affiliations:** a Research Institute of Acupuncture and Moxibustion, Shandong University of Traditional Chinese Medicine, Jinan, China; b School of Acupuncture and Massage, Shandong University of traditional Chinese Medicine, Jinan, Shandong, China.

**Keywords:** acupuncture therapy, anxiety-depressive state, IBS-D, meta-analysis, systematic review

## Abstract

**Background:**

Presently, a diverse range of Western medical interventions are accessible for the management of irritable bowel syndrome with diarrhea (IBS-D) concomitant with comorbid anxiety and depression. However, the concomitant adverse effects have also surfaced, exerting strain on healthcare resources and the socio-economic structure. In recent times, the benefits of acupuncture in the management of IBS-D with coexisting anxiety and depression have become progressively evident. Nevertheless, a paucity of evidence-based medicine exists to substantiate the utilization of acupuncture for the treatment of IBS-D with anxiety and depression. The objective of this study is to examine the effectiveness of acupuncture as an intervention for IBS-D with comorbid anxiety and depression.

**Methods:**

We searched 7 databases, including the Chinese Journal Full-text Database, Wanfang Academic Journals Full-text Database, VIP Chinese Scientific Journals Full-text Database, China Biomedical Literature Database, PubMed, Embase, and Cochrane Library, for randomized controlled trials (RCTs) related to acupuncture treatment for IBS with anxiety and depression, published from database inception to August 1, 2023. RevMan 5.4 and Stata 17.0 software were used for meta-analysis of relevant outcome measures.

**Results:**

This study included a total of 16 RCTs, involving 1305 IBS-D patients (691 in the experimental group and 614 in the control group). The meta-analysis results showed that compared to oral medication, acupuncture therapy improved HAMD scores (MD = 0.88, 95% CI = [0.68, 1.07], *P* < .00001), HAMA scores (MD = 2.32, 95% CI = [1.70, 2.93], *P* < .00001), self-rating anxiety scale scores (MD = 11.67, 95% CI = [10.85, 12.49], *P* < .00001), SDS scores (MD = 9.84, 95% CI = [8.52, 11.16], *P* < .00001), IBS-SSS scores (MD = 37.48, 95% CI = [12.17, 62.78], *P* = .004), overall response rate (MD = 1.27, 95% CI = [1.20, 1.35], *P* < .00001), and relapse rate (MD = 0.27, 95% CI = [0.16, 0.47], *P* < .00001) in patients with IBS-D comorbid with anxiety and depression.

**Conclusion:**

Acupuncture treatment has a definite and beneficial effect on IBS-D patients with comorbid anxiety and depression.

## 1. Introduction

Irritable bowel syndrome (IBS) is classified as a functional gastrointestinal disorder, distinguished by recurring abdominal pain and alterations in bowel frequency and habit, devoid of any discernible organic abnormalities. This condition is linked to a disruption in the interplay between the gastrointestinal system and the brain.^[1]^ The prevalence of IBS exhibits regional disparities, with a global average estimated at approximately 15%. Notably, China reports a prevalence ranging from 19.58% to 23.40%, while North America and Europe display a range of 10% to 25%.^[2]^ According to research findings, a significant proportion of patients seeking treatment at Chinese gastroenterology clinics receive a diagnosis of irritable bowel syndrome (IBS).^[3]^ The Rome IV criteria categorize IBS into subtypes, namely constipation-predominant (IBS-C), diarrhea-predominant (IBS-D), mixed (IBS-M), and undefined (IBS-U). Among these subtypes, IBS-D is the most prevalent, affecting approximately 40% of individuals with IBS.^[4]^ It is worth noting that individuals with IBS-D experience a lower quality of life, incur higher treatment expenses, and face an increased susceptibility to mental health disorders such as depression when compared to individuals with other subtypes.^[5]^

The precise etiology of IBS-D remains uncertain at present. Recent comprehensive investigations have implicated visceral hypersensitivity, alterations in the gut microbiota, psychological and physical stress, changes in gastrointestinal motility, and abnormalities in the gut–brain interaction as potential contributory factors to the development of IBS-D.^[6]^ The occurrence of depression (27.4%) and anxiety (38.1%) is notably elevated among individuals diagnosed with IBS-D, with a comorbidity rate of anxiety and depression reaching 23%.^[7]^ The gut–brain axis, a reciprocal neuroendocrine communication system, encompasses intricate interactions among the autonomic nervous system, the hypothalamic–pituitary–adrenal (HPA) axis, and the microbiota. This axis plays a pivotal role in establishing a significant physiological connection between IBS and the manifestation of depression and anxiety, facilitating the transmission of neural and hormonal signals between the gastrointestinal tract and the central nervous system.^[8]^ In individuals diagnosed with irritable bowel syndrome with diarrhea (IBS-D), it is possible for abnormalities to be present in the interaction between the gastrointestinal system and the brain. This can be attributed to aberrant neural regulation and heightened visceral sensation within the intestines, which can impact the brain’s centers responsible for emotional regulation through the gut–brain axis. Consequently, this can contribute to the worsening of anxiety and depression symptoms. Additionally, the occurrence of gastrointestinal symptoms and mood disorders can be influenced by the interplay of inflammation, immune system activation, and stress responses, facilitated by neural and hormonal signals within the gut–brain axis. Furthermore, dysbiosis of the gut microbiota has been observed to be linked to IBS-D, anxiety, and depression.^[9,10]^ Visceral hypersensitivity is an important physiological and pathological basis for symptoms such as abdominal pain and discomfort in IBS. Chronic pain, changes in bowel habits, or alterations in intestinal motility can lead to negative emotions and contribute to the development of anxiety and depression.^[11]^ Studies have shown that metabolites produced from tryptophan (Trp) metabolism, such as serotonin (5-HT) and kynurenine (Kyn), play an important role in sensitizing intestinal sensory nerves, intestinal motility, and intestinal mucosal barrier function, and they also have a significant impact on psychology and emotions.^[12]^

Conventional treatments for IBS-D primarily involve medication, such as antibiotics, 5-HT3 receptor antagonists (e.g., alosetron), antispasmodics (e.g., compound belladonna), antidepressants (e.g., fluoxetine), as well as dietary and lifestyle interventions.^[13]^ However, these approaches have limitations or controversies. Long-term use of medications like alosetron can lead to adverse effects such as ischemic colitis, severe constipation, and ventricular tachycardia. Long-term use of antidepressants can result in adverse consequences such as sleep disorders, movement disorders, and drug resistance.^[14,15]^ Acupuncture, a traditional Chinese therapy, has gradually gained acceptance and recognition domestically and internationally due to its simple operation, affordable cost, and overall therapeutic effectiveness.^[16]^ According to the World Health Organization, acupuncture is included as a common treatment method in the healthcare systems of more than half of its member countries.^[17]^ Acupuncture can stimulate acupoints and affect the regulation of the central nervous system, including the HPA axis and the sympathetic nervous system. These regulatory effects may help alleviate inflammatory responses, regulate gastrointestinal motility, and reduce symptoms, while also having a positive impact on anxiety and depression symptoms.^[18]^ Recent research suggests that acupuncture may exert its therapeutic effects by modulating immune system function. Acupuncture stimulation can affect the production and release of inflammatory factors, reduce the levels of interleukin-8 (IL-8) and tumor necrosis factor-alpha (TNF-α) in IBS-D patients, regulate immune cell activity, and alleviate inflammation. In IBS-D patients with comorbid anxiety and depression, changes associated with immune system abnormalities may be present, and acupuncture may improve these symptoms by modulating the immune system.^[19]^ Different studies have yielded different results. Some systematic reviews have shown that acupuncture and sham acupuncture have similar effects in improving IBS symptoms,^[20]^ while another meta-analysis indicated that acupuncture was more effective than medication.^[21]^ Therefore, we will use Cochrane systematic review methods to evaluate research quality and conduct a meta-analysis on the effects of different types of acupuncture-related therapies on anxiety and depression in IBS-D patients.

## 2. Methods

### 2.1. Search strategy

A computerized search was conducted in databases including China National Knowledge Infrastructure, WanFang Data, CBMdisc, PubMed, Web of Science, The Cochrane Library, VIP, and Embase. The search aimed to collect all relevant literature on acupuncture treatment for anxiety and depression in IBS-D patients, up until July 12, 2023. The search strategy involved a combination of subject terms and free text terms, tailored to each individual database. The search terms included acupuncture-moxibustion, acupuncture therapy, electroacupuncture, acupuncture, moxibustion, Diarrhea irritable bowel syndrome.

### 2.2. Inclusion criteria

#### 2.2.1. Study types

Randomized controlled clinical studies (RCTs) on acupuncture treatment for IBS-D.

#### 2.2.2. Study participants

Patients with a confirmed diagnosis of IBS-D and comorbid anxiety and/or depression.

#### 2.2.3. Interventions

Experimental group: Acupuncture, moxibustion, warm needle, and other acupuncture-related treatment methods; Control group: Conventional oral medication treatment.

#### 2.2.4. Outcome measures

Primary outcome measures: Self-rating depression scale (SDS), Self-rating anxiety scale (SAS), Hamilton depression scale (HAMD), and Hamilton anxiety scale (HAMA) scores.

Secondary outcome measures: Overall response rate based on treatment effectiveness, irritable bowel syndrome symptom severity scale (IBS-SSS) score, and recurrence rate.

### 2.3. Exclusion criteria

Literature without access to full-text or specific data. Duplicate publications. Conference comments or abstracts. Studies that do not report relevant indicators of anxiety and depression in IBS-D.

### 2.4. Literature selection

First, the literature screening begins by removing duplicate publications. Then, the screening is carried out based on the titles and abstracts of the articles. Subsequently, a full-text evaluation is conducted to select RCTs that meet the inclusion and exclusion criteria. For this study, the first author and second author independently perform the literature selection and data extraction in parallel. After completing the literature search, the included literature and relevant extracted data are cross-checked. A data extraction table is established using Excel 2016 to extract information from the eligible literature. The extracted information mainly includes the first author, publication date, treatment regimen, total number of participants, age, disease duration, and outcome measures. In case of any disagreements, a third-party expert is consulted for judgement and discussion.

### 2.5. Evaluation of literature quality

Two researchers assessed the methodological quality and risk bias (random sequence generation, allocation concealment, blinding method, incomplete outcome data, selective reporting, and other bias) of all included studies using the RevMan 5.4 software (Cochrane Collaboration, Oxford, UK) based on Cochrane Handbook for Systematic Reviews. Any disagreement was resolved by discussion until consensus was reached or by consulting a third researcher.

### 2.6. Statistical analysis

Review Manager 5.4 software (Cochrane Handbook) was used for statistical analysis. Results are reported as standard mean differences (MDs) in 95% confidence intervals (95%CI) for continuous outcomes and 95% CI for relative risk (RR) for dichotomous outcomes. Statistical heterogeneity among studies was analyzed by *I*^2^ test and chi-square test. In the absence of heterogeneity, we used a fixed-effects model (*P* > .05 by the chi-square test and *P* < 50% by the *I*^2^ test). Sensitivity analysis was conducted to examine the influence of individual studies on the stability of the meta-analysis results. For continuous variables such as SAS, SDS, HAMA, HAMD, and IBS-SSS scores in outcome measures, MD and standard deviation (SD) are used. For binary variables such as overall response rate, RR and 95%CI are used. In cases where the data is not normally distributed, the median, P25, and P75 values are transformed using the method described by McGrath.^[22]^ The median, maximum value, and minimum value are transformed using the method described by Hozo.^[23]^ When the number of included studies is >10, a funnel plot is used to assess publication bias.

## 3. Results

### 3.1. Literature search

**Figure 1.**
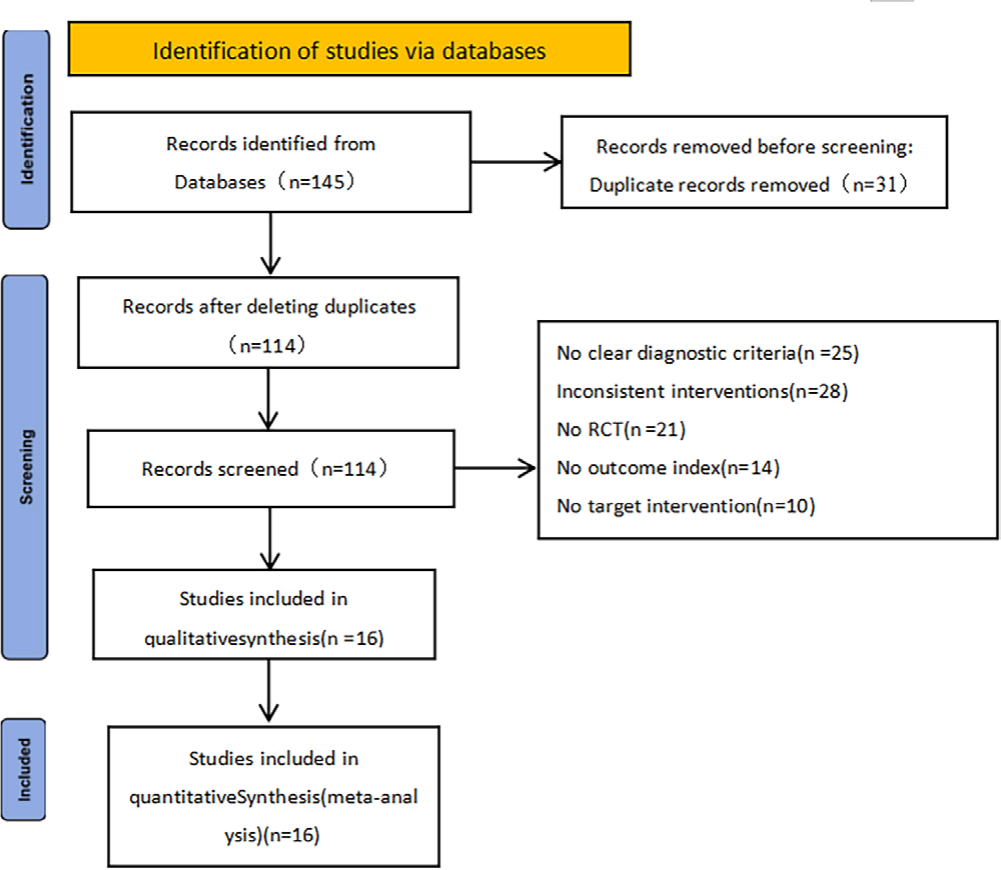
Flow diagram.

After the initial screening, a total of 145 relevant articles were identified, including 121 Chinese articles and 24 English articles. After removing 31 duplicate articles, 81 articles were excluded based on their titles and abstracts. After a full-text assessment, an additional 17 articles were excluded. Finally, a total of 16 articles^[24–39]^ were included in the study. The detailed process of literature inclusion is shown in Figure 1.

### 3.2. The basic characteristics of included studies

As shown in Table 1, a total of 16 articles were included, including 15 Chinese articles^[24–38]^ and 1 English article.^[39]^ These studies involved 1308 participants (693 in the experimental group and 615 in the control group). Seven studies^[24,25,29–31,36,37]^ included the Hamilton anxiety rating scale (HAMA) and the Hamilton depression rating scale (HAMD), 8 studies ^[26–28,32–35,38]^ included the self-rating anxiety scale (SAS), 7 studies ^[26–28,32,34,35,39]^ included the self-rating depression scale (SDS), 5 studies^[25,28–30,39]^ included the irritable bowel syndrome severity scoring system (IBS-SSS), and 13 studies^[25–32,34–36,38,39]^ included the overall effective rate (Table 1).

**Table 1:** Summary of the included study characteristics.

References	Age (mean ± sd)/range	n	Course of disease/month(mean ± sd)/range	Treatment	Course	Duration of acupuncture/min	Outcome measures
Yang 2022^[24]^	I:32.43 ± 6.76	I: (30)	I: (44.52 ± 37.8)	ACU + MOX	3W	30 min	③④
	C:33.67 ± 4.69	C: (30)	C: (46.68 ± 35.64)	WM			
Wei 2023^[25]^	I:35.62 ± 5.59	I: (43)	I: (35.62 ± 5.59)	ACU + WM	4W	30 min	③④⑤⑥
	C:35.43 ± 5.71	C: (43)	C: (32.98 ± 2.56)	WM			
Bu 2020^[26]^	I:34.2 ± 5.2	I: (46)	I: (34.2 ± 5.2)	ACU + WM	5W	30 min	①②⑥
	C:34.1 ± 5.2	C: (46)	C: (34.1 ± 5.2)	WM			
Jia 2022^[27]^	I:37.80 ± 11.94	I: (30)	I: (37.80 ± 11.94)	ACU + CH	4W	30 min	①②⑥
	C:38.83 ± 12.37	C: (30)	C: (25.8 ± 10.92)	CH			
Li 2022^[28]^	I:37.3 ± 9.12	I: (47)	I: (37.3 ± 9.12)	ACU + MOX	2W	30 min	①②⑤⑥⑦
	C:40.8 ± 9.78	C: (46)	C: (40.8 ± 9.78)	WM			
Sun 2022^[29]^	I:36.2 ± 5.2	I: (40)	I: (36.2 ± 5.2)	ACU + CH	4W	30 min	③④⑤⑥
	C:35.8 ± 5.5	C: (40)	C: (35.8 ± 5.5)	WM			
Chen 2021^[30]^	I:41 ± 6	I: (31)	I: (22.56 ± 5.64)	ACU + MOX	6W	25 min	③④⑤⑥⑦
	C:39 ± 7	C: (30)	C: (24.24 ± 3.48)	WM			
Yang 2020^[31]^	I:54.0 ± 6.3	I: (20)	I: (48 ± 39.6)	ACU + MOX	2W	30 min	③④⑥
	C:54.0 ± 6.1	C: (20)	C: (48 ± 43.2)	WM			
Li 2018^[32]^	I:41 ± 9	I: (43)	I: (10.98 ± 5.12)	ACU + MOX + CH	6W	30 min	①②⑥
	C:41 ± 9	C: (43)	C: (10.79 ± 5.04)	CH			
Cheng 2023^[33]^	I:45.4 ± 8.1	I: (30)	I: (76.44 ± 31.44)	ACU + CH	4W	After air conduction, the needle was removed	①
	C:44.6 ± 7.9	C: (30)	C: (79.2 ± 35.4)	CH			
Zhang 2022^[34]^	I:37.22 ± 9.25	I: (40)	I: (56.64 ± 108.6)	MOX + CH	4W	N	①②⑥⑦
	C:37.89 ± 8.65	C: (40)	C: (55.2 ± 106.08)	WM			
Liao 2020^[35]^	I:37.91 ± 2.92	I: (35)	I: (56.31 ± 5.55)	MOX	2W	40 min	①②⑥⑦
	C:38.46 ± 3	C: (35)	C: (55.86 ± 4)	WM			
Zhou 2014^[36]^	I:39.3	I: (45)	I: (75.6)	ACU + MOX + CH	8W	N	③④⑥
	C:38.9	C: (45)	C: (79.2)	WM			
Chen 2012^[37]^	I:41. 90 ± 10.01	I: (34)	I: (98.4 ± 56.88)	ACU	4W	30 min	③④⑥⑦
	C:40. 50 ± 8. 75	C: (30)	C: (105.36 ± 61.32)	WM			
Han 2013^[38]^	I:41 ± 11.12	I: (144)	I: (39 ± 29.4)	ACU + CH	8W	20 min	①⑥
	C:40 ± 10. 72	C: (72)	C: (48.48 ± 25.92)	WM			
Meng 2019^[39]^	I:39.3 ± 11.5	I: (35)	I: (25.9 ± 12.0)	ACU	4W	30 min	②⑤⑥
	C:38.4 ± 13.5	C: (35)	C: (26.0 ± 12.9)	WM			

ACU = acupuncture, C = Control, CH = Chinese herb medicine, I = intervention, M = month, MOX = moxibustion, NR = no reported, W = week, WM = Western medicine of anti-diarrheal or antispasmodic.

①, SAS; ②, SDS; ③, HAMA; ④, HAMD; ⑤, IBS-SSS; ⑥, Overall effective rate; ⑦, recurrence rate.

### 3.3. The quality assessment of included studies

**Figure 2.**
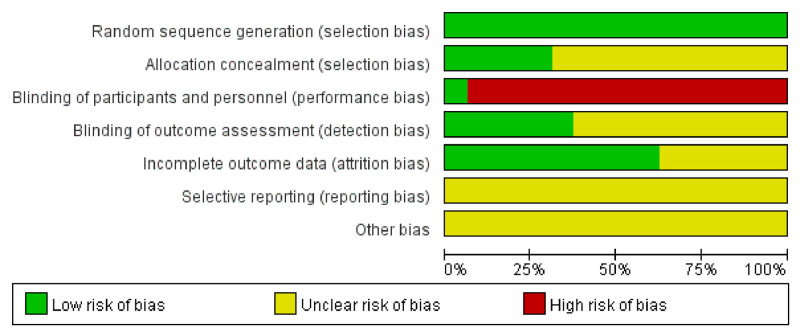
Risk of bias graph.

**Figure 3.**
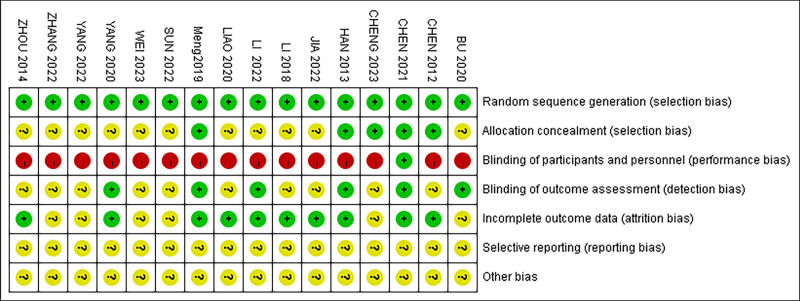
Risk of bias summary.

Eleven studies^[24,25,27–30,32,34,35,37,39]^ used a random number table method for randomization, while the remaining 5 studies^[26,31,33,36,38]^ mentioned randomization without providing detailed information about the randomization method. Due to the nature of acupuncture, it was challenging to implement blinding during the treatment, and no study reported allocation concealment and blinding. Fourteen studies^[24–32,35–39]^ reported missing data and were considered at low risk in terms of completeness. The assessment of bias risk is shown in Figures 2 and 3.

### 3.4. Meta-analysis

#### 3.4.1. The Hamilton depression scale score

**Figure 4.**
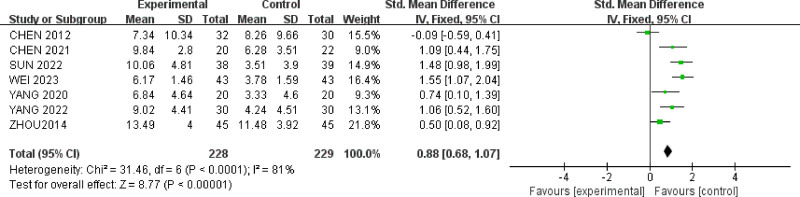
Forest plot of HAMD scores for acupuncture treatment in IBS-D with comorbid anxiety and depression.

Seven studies^[24,25,29–31,36,37]^ reported HAMD scores for a total of 457 patients. There was significant heterogeneity among the studies (*P* < .0001, *I*^2^ = 81%). Sensitivity analysis using the leave-one-out method still showed substantial heterogeneity (Table 2). Acupuncture therapy (MD = 0.88, 95% CI = [0.68, 1.07]) was found to be more effective than oral medication in reducing HAMD scores for IBS-D patients with anxiety and depression (*P* < .00001) (Fig. 4).

**Table 2:** Sensitivity analysis of HAMD scores for acupuncture combined with medication therapy in included studies.

Exclude literature	MD (95% CI)	*P*	*I*^2^ (%)
Zhou 2014	0.98 (0.76, 1.20)	<.0001	82
Sun 2022	0.77 (0.56, 0.98)	<.0001	80
Wei 2023	0.75 (0.53, 0.96)	<.0001	78
Yang 2020	0.89 (0.69, 1.10)	<.0001	84
Yang 2022	0.85 (0.64, 1.06)	<.0001	84
Chen 2021	0.86 (0.65, 1.06)	<.0001	84
Chen 2012	1.05 (0.84, 1.27)	<.0001	65

#### 3.4.2. *The* Hamilton anxiety scale *score*

**Figure 5.**
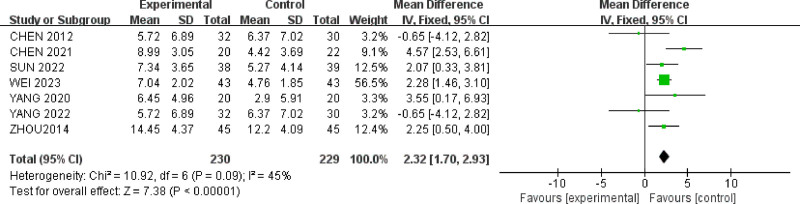
Forest plot of HAMA scores for acupuncture treatment in IBS-D with comorbid anxiety and depression.

Seven studies^[24,25,29–31,36,37]^ reported HAMA scores for a total of 459 patients (Fig. 5). There was relatively low heterogeneity among the studies (*P* = .09, *I*^2^ = 45%), and a fixed-effect model was used. The results showed that acupuncture therapy (MD = 2.32, 95% CI = [1.70, 2.93]) was more effective than oral medication in reducing HAMA scores for IBS-D patients with anxiety and depression(*P* < .00001).

#### 3.4.3. *The* self-rating anxiety scale *score*

**Figure 6.**
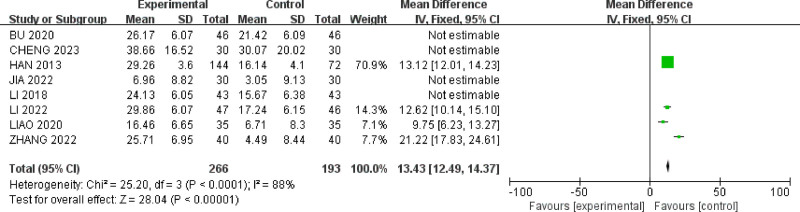
Forest plot of self-rating anxiety scale scores for acupuncture treatment in IBS-D with comorbid anxiety and depression.

Eight studies^[26–28,32–35,38]^ reported SAS scores for a total of 757 patients. There was substantial heterogeneity among the studies (*P* < .00001, *I*^2^ = 92%) (Fig. 6). A random-effects model was used, and sensitivity analysis was conducted by excluding 1 acupuncture study at a time (Table 3), but the heterogeneity remained significant. Meta-analysis showed that acupuncture therapy (MD = 11.67, 95% CI = [10.85, 12.49]) was more effective than oral medication in reducing SAS scores for IBS-D patients with anxiety and depression (*P* < .00001). Please refer to Figure 6 for details.

**Table 3:** Sensitivity analysis of acupuncture therapy in included studies for SAS scores.

Exclude literature	MD (95% CI)	*P*	*I*^2^ (%)
Bu 2020	12.51 (11.64, 13.37)	<.00001	89
Zhang 2022	11.08 (10.24, 11.92)	<.00001	89
Li 2018	12.01 (11.15, 12.87)	<.00001	92
Cheng 2023	11.69 (10.88, 12.51)	<.00001	93
Jia 2022	11.93 (11.10, 12.76)	<.00001	92
Han 2013	10.00 (8.80, 11.19)	<.00001	92
Li 2022	11.56 (10.69, 12.42)	<.00001	93
Liao 2020	11.78 (10.94, 12.62)	<.00001	93

#### 3.4.4. The self-rating depression scale score

**Figure 7.**
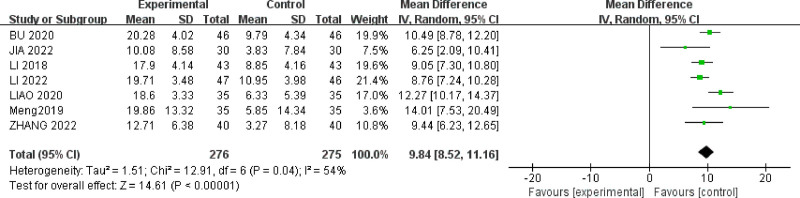
Forest plot of SDS scores for acupuncture treatment in IBS-D with comorbid anxiety and depression.

**Figure 8.**
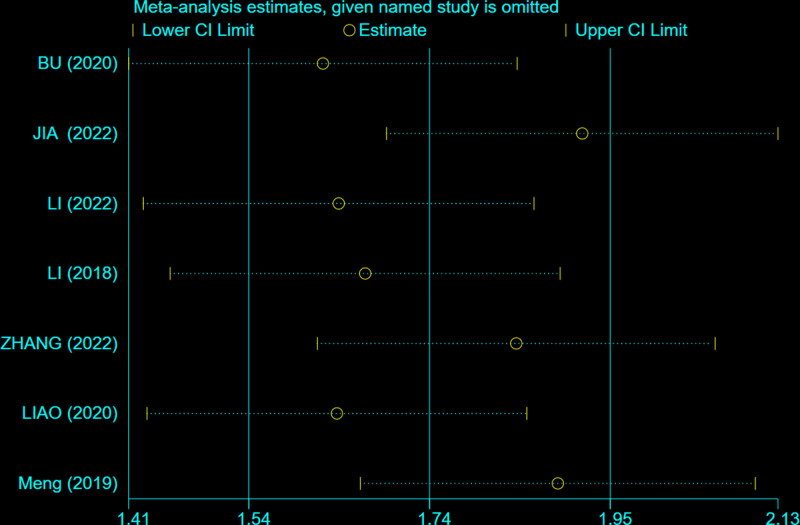
Sensitivity analysis of acupuncture therapy in included studies for SDS scores.

Seven studies^[26–28,32,34,35,39]^ reported SDS scores for a total of 55 IBS-D patients. There was moderate heterogeneity among the studies (*P* = .04, *I*^2^ = 54%) (Fig. 7). A random-effects model was used, and sensitivity analysis was conducted on the acupuncture studies. As shown in Figure 8, the study by Jia Xiaomeng^[27]^ had a significantly larger effect size compared to the other 2 studies. After excluding this study, the heterogeneity among the remaining studies decreased (*P* = .07, *I*^2^ = 50%).The results indicated that acupuncture therapy (MD = 9.84, 95% CI = [8.52, 11.16]) was more effective than oral medication in reducing SDS scores for IBS-D patients with anxiety and depression(*P* < .00001).

#### 3.4.5. *The* irritable bowel syndrome symptom severity scale *score*

**Figure 9.**
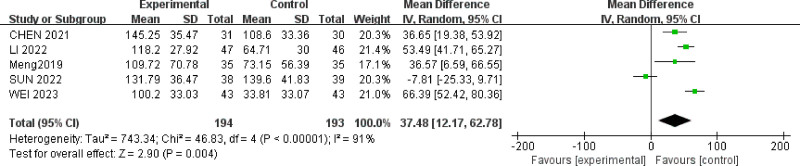
Forest plot of IBS-SSS scores for acupuncture treatment in IBS-D with comorbid anxiety and depression.

Five studies^[25,28–30,39]^ reported IBS-SSS scores for a total of 387 IBS-D patients. However, there was high heterogeneity among the studies (*P* < .00001, *I*^2^ = 91%) (Fig. 9). A random-effects model was used, and sensitivity analysis was conducted by excluding 1 study at a time (Table 4). Even after each exclusion, the heterogeneity remained significant. Meta-analysis results showed that acupuncture therapy and combination therapy (MD = 37.48, 95% CI = [12.17, 62.78]) were more effective than oral medication in reducing IBS-SSS scores for IBS-D patients with anxiety and depression (*P* = .004).

**Table 4:** Sensitivity analysis of acupuncture therapy in included studies for IBS-SSS scores.

Exclude literature	MD (95% CI)	*P*	*I*^2^ (%)
Sun 2022	50.66 (36.96, 64.37)	<.00001	63
Wei 2023	29.79 (0.80, 58.78)	.04	91
Chen 2021	37.56 (5.22, 69.90)	.02	94
Li 2022	33.06 (−1.42, 67.54)	=.06	93
Meng 2019	37.62 (8.34, 66.90)	=.01	94

#### 3.4.6. Overall effectiveness rate

**Figure 10.**
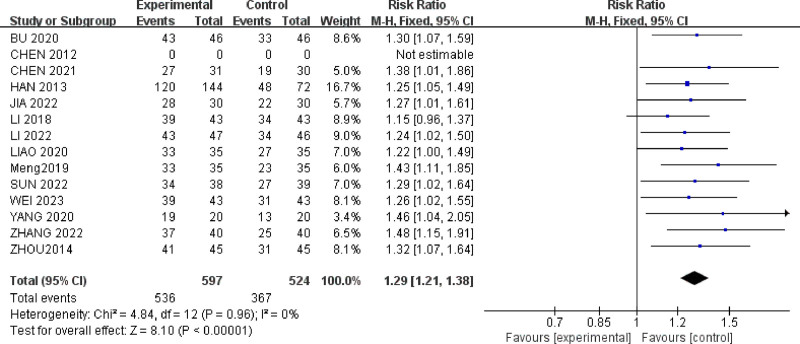
Forest plot of Overall effectiveness rate for acupuncture treatment in IBS-D with comorbid anxiety and depression.

Fourteen studies^[25–32,34–39]^ reported the overall response rate for a total of 1185 IBS-D patients. There was minimal heterogeneity among the studies (*P* = .66, *I*^2^ = 0%) (Fig. 10). A fixed-effect model was used for meta-analysis. The results showed that acupuncture therapy and combination therapy (MD = 1.27, 95% CI = [1.20, 1.35]) were more effective than oral medication in reducing the overall response rate for IBS-D patients with anxiety and depression (*P* < .00001).

#### 3.4.7. Recurrence rate

**Figure 11.**
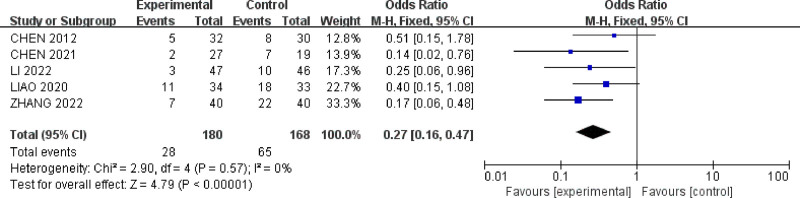
Forest plot of recurrence rate for acupuncture treatment in IBS-D with comorbid anxiety and depression.

Five studies^[28,30,34,35,37]^ reported the relapse rate for a total of 348 IBS-D patients. There was minimal heterogeneity among the studies (*P* = .57, *I*^2^ = 0%) (Fig. 11). A fixed-effect model was used for meta-analysis. The results showed that acupuncture therapy (MD = 0.27, 95% CI = [0.16, 0.47]) was more effective than oral medication in reducing the relapse rate for IBS-D patients with anxiety and depression (*P* < .00001).

### 3.5. Publication bias

Since the number of studies included for each outcome in this study is <10, a funnel plot will not be generated.

### 3.6. Frequency statistics of acupoints

Among the 16 included articles, a total of 16 acupuncture prescriptions were identified. The frequency of acupoint usage was calculated (Table 5). A total of 23 acupoints were involved, with a cumulative frequency of 103. The top 5 acupoints in terms of frequency were Tianshu (ST25), Zusanli (ST36), Sanyinjiao (SP6), Shangjuxu (ST37), and Taichong (LR3).

**Table 5:** Frequency statistics of acupoint usage in included studies.

Serial number	Acupoint	Frequency	Rate	Serial number	Acupoint	Frequency	Rate
1	Tianshu (ST25)	14	13.60%	13	Pishu (BL20)	2	1.94%
2	Zusanli (ST36)	12	11.65%	14	Shenshu (BL23)	2	1.94%
3	Sanyinjiao (SP6)	10	9.70%	15	Dachangshu (BL25)	2	1.94%
4	Shangjuxu (ST37)	10	9.70%	16	Guanyuan (RN4)	1	0.97%
5	Taichong (LR3)	10	9.70%	17	Shuidao (ST28)	1	0.97%
6	Neigaun (PC6)	6	5.82%	18	Yinlingquan (SP9)	1	0.97%
7	Shenque (RN8)	6	5.82%	19	Fuliu (KI7)	1	0.97%
8	Yintang (EX-HN3)	6	5.82%	20	Ganshu (BL18)	1	0.97%
9	Baihui (DU20)	6	5.82%	21	Shenmen (HT7)	1	0.97%
10	Zhongwan (RN12)	4	3.88%	22	Qihai (CV6)	1	0.97%
11	Shenting (DU24)	3	2.91%	23	Weishu (BL21)	1	0.97%
12	Sishencong (EX-HN1)	2	1.94%				

## 4. Discussion

This study included 16 RCTs and conducted a meta-analysis to compare the efficacy and safety of acupuncture therapy for treating IBS-D with comorbid anxiety and depression. The results showed that acupuncture or acupuncture combined with herbal medicine was more effective than medication in improving SDS scores, SAS scores, HAMD scores, HAMA scores, overall response rate, IBS-SSS scores, and relapse rate. Additionally, acupuncture treatment demonstrated a higher level of safety. It should be noted that there was considerable heterogeneity in some outcome measures in this study, which may be attributed to differences in patient characteristics, acupuncture points selection, treatment duration, and methods across different studies. Therefore, individual factors and specific circumstances should be taken into consideration when making treatment decisions.

This study utilized the SAS and HAMA scales to assess patients’ anxiety levels, and the SDS and HAMD scales to assess their depression levels. The SAS and SDS are self-report questionnaires that rely on patients’ subjective perceptions, where individuals choose the answers that best match their own experiences to evaluate their anxiety and depression levels. The advantages of these scales lie in their simplicity, ease of use, and practicality. On the other hand, the HAMA and HAMD scales involve clinical observers or doctors assessing patients’ anxiety and depression levels. These scales typically consist of multiple items, each with specific descriptions and rating criteria, allowing doctors to evaluate patients based on their behaviors, emotional expressions, and other factors. The HAMA and HAMD scales offer advantages in terms of evaluation precision and objectivity, providing more detailed and accurate assessment outcomes. By employing both self-report scales and other assessment scales in the research, information on patients’ anxiety and depression can be obtained from different perspectives. Self-report scales reflect patients’ subjective experiences, while other assessment scales provide more objective and detailed evaluations. The comprehensive use of different types of scales allows for a more comprehensive assessment of patients’ psychological status and provides deeper insights for research purposes.

The treatment groups in all 16 studies used traditional acupuncture therapy. The frequency statistics of selected acupoints included in the studies showed that Tianshu (ST25), Zusanli (ST36), Sanyinjiao (SP6), Shangjuxu (ST37), and Taichong (LR3) are the acupoints with higher frequencies in acupuncture treatment for IBS-D with comorbid anxiety and depression. Neurotrophic Growth Factor (NGF) is distributed in organs such as the brain, nerve ganglia, and intestines. It is a neurotrophic factor that plays an important role in neuronal development, survival, and maintenance of function. NGF can modify the plasticity of local neurons, promote neuronal growth and connections, and influence the distribution of sensory nerve endings and the expression of Transient Receptor Potential Vanilloid 1 (TRPV1) protein. The mechanism of action of NGF mainly involves binding to specific receptors, such as the TrkA receptor, to activate downstream signaling pathways that affect cell growth, survival, and function. In the gastrointestinal tract, NGF can increase visceral hypersensitivity in the gastrointestinal tract, leading to discomfort such as abdominal pain.^[40]^ The dysfunction of intestinal function is associated with abnormal expression of Brain-derived neurotrophic factor (BDNF) and CAMP-response element-binding protein (CREB). Animal experiments have shown that electro-acupuncture on Tianshu (ST25) and Shangjuxu (ST37) can improve visceral hypersensitivity in an IBS-D rat model by reducing the protein expression of NGF/TrkA/TRPV1 in colonic tissue, as well as reducing the expression of RHOA/ROCK, NF-κB, BDNF, and CREB, and increasing the expression of SIRT2 to reduce the inflammation response and intestinal dysfunction caused by the model. At the same time, it can improve the depressive symptoms associated with IBS-D by reversing the abnormal expression of NF-κB and SIRT2 in the hippocampal tissue of IBS-D model rats, thus exerting a bidirectional regulatory effect.^[41]^ Research has found that various mediators, such as histamine, serotonin, and prostaglandins, secreted by colonic mast cells in IBS-D patients can lead to abnormal inflammatory responses and neural transmission in the intestines. The excessive release of these chemicals may increase intestinal permeability, leading to intestinal barrier dysfunction. The compromised intestinal barrier allows harmful substances and bacteria to penetrate the intestinal wall, stimulating the intestinal immune system and causing an inflammatory response, further exacerbating the symptoms of IBS-D.^[42]^ Cholecystokinin (CCK) and substance P (SP) can promote degranulation of intestinal mast cells, leading to the production of large amounts of tryptase (TPS), adenosine triphosphate (ATP), and other substances, exacerbating inflammatory reactions and damaging the intestinal barrier.^[43]^ Experimental studies have shown that electro-acupuncture on Zusanli (ST36), Tianshu (ST25), and Taichong (LR3) can downregulate the levels of CCK, SP, TPS, and ATP in the intestine, protect the intestinal barrier, and alleviate symptoms such as visceral hypersensitivity and diarrhea in IBS-D rats.^[44]^

This study has the following limitations. Firstly, the generation of random sequences may not have been adequately randomized or the randomization process was not clearly described, which could lead to an imbalance in the allocation of the study and control groups. This can affect the reliability and comparability of the results. Secondly, there was a lack of blinding: In acupuncture treatment, it is challenging to achieve complete blinding as both the therapist and the subjects can perceive the acupuncture procedure. The lack of blinding may introduce interference from treatment effects and placebo effects, thereby affecting the interpretation and comparison of the results. Thirdly, there was a lack of long-term efficacy data. In acupuncture treatment studies, if long-term follow-up data are lacking, it is difficult to accurately assess the sustained effect and potential side effects of the treatment method. The duration of follow-up is closely related to the assessment of treatment outcomes. Without long-term observation of patients and their relapse rates, we will not be able to accurately understand the long-term efficacy of acupuncture treatment for IBS-D with comorbid anxiety and depression. Therefore, in future research, it is important to strengthen long-term follow-up of acupuncture and herbal medicine combination therapy, including observation of relapse rates when evaluating treatment efficacy. This will contribute to a more comprehensive and accurate assessment of the practical effectiveness and safety of the treatment method.

The results of the meta-analysis indicate that acupuncture therapy has shown significant benefits in treating IBS-D patients with comorbid anxiety and depression, without increasing the risk of adverse events. This finding provides strong support for acupuncture as a viable treatment option. However, in order to comprehensively and reliably evaluate the effectiveness of acupuncture therapy in IBS-D treatment, large-scale and well-designed RCTs are still needed. Such study designs can reduce the occurrence of bias and improve the reliability and generalizability of the research findings. Additionally, the collection of long-term follow-up results is crucial for assessing treatment outcomes and sustainability. Therefore, future research should emphasize long-term follow-up to evaluate the long-term efficacy of acupuncture therapy in IBS-D treatment. In conclusion, although the results of the meta-analysis demonstrate the beneficial effects of acupuncture therapy in treating IBS-D with comorbid anxiety and depression, further confirmation and dissemination of these findings require more high-quality research, particularly large-scale and well-designed RCTs, along with evaluations incorporating long-term follow-up results.

## Author contributions

**Investigation:** Hongwei Sun.

**Methodology:** Yi Hou.

**Project administration:** Hongwei Sun, Zhengwen Wang.

**Resources:** Zhen Wang, Zhengwen Wang.

**Software:** Zhen Wang, Haiyan Zhang.

**Writing – review & editing:** Yi Hou, Haiyan Zhang.
